# The Role of N and S Doping on Photoluminescent Characteristics of Carbon Dots from Palm Bunches for Fluorimetric Sensing of Fe^3+^ Ion

**DOI:** 10.3390/ijms23095001

**Published:** 2022-04-30

**Authors:** Aphinan Saengsrichan, Chaiwat Saikate, Peeranut Silasana, Pongtanawat Khemthong, Wanwitoo Wanmolee, Jakkapop Phanthasri, Saran Youngjan, Pattaraporn Posoknistakul, Sakhon Ratchahat, Navadol Laosiripojana, Kevin C.-W. Wu, Chularat Sakdaronnarong

**Affiliations:** 1 Department of Chemical Engineering, Faculty of Engineering, Mahidol University, 25/25 Putthamonthon 4 Road, Salaya, Putthamonthon, Nakhon Pathom 73170, Thailand; saengsrichan.a@gmail.com (A.S.); chaiwat.saikate@gmail.com (C.S.); peeranut.silasana@gmail.com (P.S.); pattaraporn.pos@mahidol.edu (P.P.); sakhon.rat@mahidol.edu (S.R.); 2 National Nanotechnology Center (NANOTEC), National Science and Technology Development Agency (NSTDA), Pathum Thani 12120, Thailand; pongtanawat@nanotec.or.th (P.K.); wanwitoo.wan@nanotec.or.th (W.W.); jakkapop.pha@ncr.nstda.or.th (J.P.); saran.you@nanotec.or.th (S.Y.); 3 The Joint Graduate School of Energy and Environment, King Mongkut’s University of Technology Thonburi, 126 Pracha Uthit Road, Bang Mot, Thung Khru, Bangkok 10140, Thailand; navadol_l@jgsee.kmutt.ac.th; 4 Department of Chemical Engineering, National Taiwan University, No.1, Sec.4 Roosevelt Road, Taipei 10617, Taiwan; kevinwu@ntu.edu.tw; 5 Center of Atomic Initiative for New Materials (AI-MAT), National Taiwan University, Taipei 10617, Taiwan; 6 International Graduate Program of Molecular Science and Technology, National Taiwan University (NTU-MST), Taipei 10617, Taiwan

**Keywords:** carbon dots, hydrothermal, biomass, surface passivation, nitrogen and sulfur doping, photoluminescence, metal detection, fluorimetric sensing, Fe(III) fluorescence turn-off sensor, ferric ion

## Abstract

This work aims to enhance the value of palm empty fruit bunches (EFBs), an abundant residue from the palm oil industry, as a precursor for the synthesis of luminescent carbon dots (CDs). The mechanism of fIuorimetric sensing using carbon dots for either enhancing or quenching photoluminescence properties when binding with analytes is useful for the detection of ultra-low amounts of analytes. This study revealed that EFB-derived CDs via hydrothermal synthesis exceptionally exhibited luminescence properties. In addition, surface modification for specific binding to a target molecule substantially augmented their PL characteristics. Among the different nitrogen and sulfur (N and S) doping agents used, including urea (U), sulfate (S), *p*-phenylenediamine (P), and sodium thiosulfate (TS), the results showed that PTS-CDs from the co-doping of *p*-phenylenediamine and sodium thiosulfate exhibited the highest PL properties. From this study on the fluorimetric sensing of several metal ions, PTS-CDs could effectively detect Fe^3+^ with the highest selectivity by fluorescence quenching to 79.1% at a limit of detection (LOD) of 0.1 µmol L^−1^. The PL quenching of PTS-CDs was linearly correlated with the wide range of Fe^3+^ concentration, ranging from 5 to 400 µmol L^−1^ (R^2^ = 0.9933).

## 1. Introduction

Metal ions such as iron, copper, zinc, and selenium are crucial elements in biological systems that fulfill and play a vital role in several pathological and physiological processes, consisting of enzyme catalysis, oxygen transport, RNA synthesis, cellular metabolism, etc. [1,2,3]. The precise sensing of metal ions leads to more accurate diagnosis of some severe noncommunicable diseases such as Alzheimer’s disease. There is evidence that iron, even in trace amounts, can cause the presence of magnetite in the brains of people affected by Alzheimer’s. The magnetite found in the abnormal protein clusters known as amyloid plagues is symptomatic of this disease, and the putative role of magnetite is to cause neuro-generative diseases [4]. Several analytical techniques have been established to determine the concentration of Fe^3+^, including UV-vis spectrophotometry [5,6], atomic absorption spectrometry (AAS) [7], and inductively coupled plasma mass spectrometry (ICP-MS) [8,9]. However, these techniques require laborious sample preparation procedures, e.g., complexation and digestion, as well as sophisticated instrumentations, which have hindered their practical applications to in situ and on-time detection.

Lately, the fluorescence assays using transition metal chalcogenide-based quantum dots (QDs) or fluorophores have been found as an effective and sensitive sensing approach providing high sensitivity and rapid response for the detection of metal ions and organic analytes using the excitation of light at a specific wavelength [1,5,9]. Additionally, this technique is simple and more applicable if one can adjust the excitation wavelength to near-visible light, which is more healthful and requires only an unsophisticated light source such as light-emitting diode [10]. For this reason, most recently, carbon quantum dots (CQDs), which are a new class of QD having high biocompatibility, nontoxicity, and high sensitivity for nanocatalytic reactions, have emerged and been utilized as exceptional fluorophores [11]. It is claimed that carbon dots (CDs) have intrinsic abilities regarding tunable fluorescence excitation and emission properties and high surface functionality to enhance their affinity toward metal ions. Thus, numerous efforts have been made on the functionalization and passivation of organic molecules at the edge or heteroatom incorporated into CD structures for energy and sensing applications [12,13]. Most recently, CDs conjugated with metals, e.g., lanthanides (e.g., Tb [14]), were reported for their ratiometric temperature sensing ability.

It was reported that the optical properties of CDs for both emission and excitation wavelengths, as well as fluorescence intensity, could be adjusted by doping with some elements [15], such as nitrogen (N-doped), sulfur (S-doped), phosphorus (P-doped), boron (B-doped), or a combination of these elements called heteroatom doping. The influence of doping elements on CDs is due to the alteration of orbital energy levels between the lowest unoccupied molecular orbitals, or LUMO, and the highest occupied molecular orbitals, or HOMO. Electron charges are randomly distributed in the doped CDs. The electrons in the HOMO can be excited to the LUMO, which enhances the CD fluorescence [16]. This means that doped CDs can be employed for highly sensitive sensing for the detection of metal ions.

The precise monitoring of biological pathways, which is a very important diagnosis strategy, necessitates the detection of Fe^3+^ at low concentration levels. Therefore, in this research, we aim to study the ability of CDs derived from oil palm empty fruit bunches (EFBs) to detect metal ions. Homoatom CDs with oxygen functionalization using a one-pot hydrothermal approach are first used. Afterward, the influence of various types of nitrogen (N) dopants (i.e., urea and *p*-phenylenediamine), sulfur (S) dopants (i.e., sulfate and thiosulfate), and different concentrations of N and S dopants on optical and structural properties of CDs are investigated. Fluorimetric sensing of metal ions using NS/CDs is conducted at a very low concentration of metal ions, and the results from the kinetic study are discussed accordingly.

## 2. Results and Discussion

### 2.1. Synthesis and Characterization of CDs under Hydrothermal Conditions and Effect of Different Dopants on CD Properties

**Figure 1:**
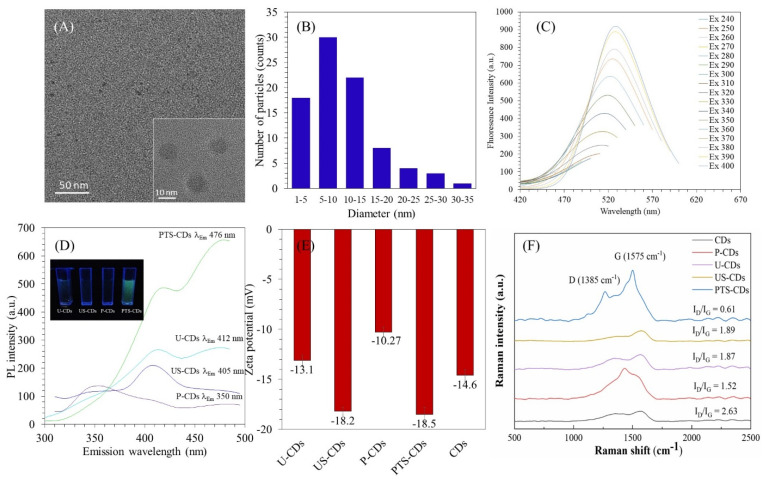
(**A**) HRTEM images of CDs synthesized at 220 °C for 6 h under hydrothermal conditions at ×30,000 magnification; inset is CDs at ×200,000 magnification; (**B**) size distribution of CDs; (**C**) PL property of CDs; (**D**) PL intensity of nitrogen-doped and nitrogen-and-sulfur-doped CDs, namely U-CDs, US-CDs, P-CDs, and PTS-CDs (inset is a photo of CD solution under UV light at 366 nm wavelength); (**E**) zeta potential of CDs and surface passivated CDs; and (**F**) Raman spectra of PTS-CDs, P-CDs, and CDs.

The synthesis of CDs from EFBs using a hydrothermal reaction was successfully conducted at 220 °C for 6 h. After careful separation and purification, CDs having spherical morphologies with an average diameter of 10.3 ± 3.1 nm were obtained, as demonstrated in Figure 1A,B. As shown in Figure 1C, the CDs exhibited excellent excitation-independent PL characteristics between the excitation wavelengths of λ_Ex_ = 240 nm and 360 nm. The maximum emission wavelength was demonstrated at λ_Em_ = 424 nm when excited at λ_Ex_ = 340 nm. After surface passivation using various types of nitrogen or nitrogen and sulfur atoms by using one-pot synthesis, dramatically enhanced PL intensity of the CDs was achieved, particularly when the nitrogen passivating agent was a mixture of *p*-phenylenediamine (P), and the sulfur passivating agent was sodium thiosulfate (TS), named as PTS-CDs (Figure 1D). A two-fold PL intensity of the PTS-CDs was obtained when compared with neat CDs and U-CDs and more than three-fold PL intensity of urea sulfate (US-CDs) and solely *p*-phenylenediamine (P-CDs). The inset of Figure 1D demonstrates that CDs doped with a mixture of p-phenylenediamine and sodium thiosulfate (PTS-CDs) exhibited the strongest PL intensity with a green fluorescent emission (λ_Em_ = 476 nm) from the excitation wavelength at 260 nm, which corresponded to a maximum QY of 48%, while neat CDs gave 39% QY. It was reported that CD defects due to the passivation process could provide heteroatoms acting as excitation energy traps and have an impact on the photoluminescence of CDs. Particularly, the sulfur atoms in CDs were found to play a crucial role as catalysts for an oxidation-reduction reaction; thus, the presence of a sulfur heteroatom presumably introduces more dense, passivated surface defects on CDs and further enhances CD photoluminescence. Therefore, PTS-CDs are promising and highly sensitive photonic sensing agents for further application using either a turn-on or turn-off fluorescent performance of CDs.

Surface passivation was also indicated by zeta potential, which represents the density of surface charges of CDs. The stability of CDs in an aqueous solution without aggregation can also be demonstrated by zeta potential. The more value shown, the better stability and greater electrostatic repulsion force among each nanoparticle. As shown in Figure 1E, the zeta potential of every sample exhibited negative-charged values mainly due to hydroxyl groups and carboxylic groups on the surface of the material [17]. Considering in detail, the zeta potential of the CDs was −14.6 mV, and a reduction in the negative zeta potential was observed for nitrogen-doped CDs, e.g., U-CDs (−13.10 mV) and P-CDs (−10.27 mV). The reason was due to the balance of positive charges derived from amine groups in urea and *p*-phenylenediamine, which caused a net balance of charges at a neutral state, resulting in fewer negatively charged U-CDs and P-CDs on the surface. In contrast, an increase of negative surface charges was achieved in the case of PNa-CDs, and US-CDs in which thiosulfate (S_2_O_3_^2−^) and sulfate (SO_4_^2−^) triggered −18.5 and −18.2 mV zeta potentials, respectively. The results showed a good agreement with the values previously reported for carboxylic acid (COOH)-coated CDs [18].

From HRTEM image analysis of surface-passivated CDs, which were US-CDs and PTS-CDs, as shown in Figure S1, US-CDs and PTS-CDs had identically spherical shapes with a narrow range of diameter similar to CDs (Figure 1A,B). All the synthesized materials were uniform and distributed without aggregation. However, a significant change in the average diameter and morphology of US-CDs and PTS-CDs compared with neat CDs synthesized from EFBs via hydrothermal reaction was found. The average diameters of US-CDs and PTS-CDs decreased substantially compared to CDs. The reason was mainly due to the oxygen reduction enhancement of the precursor due to an amine group. Thus, a highly graphitic carbon structure was obtained after urea and sulfate doping (US-CDs), and an even more graphitic structure when doping with *p*-phenylenediamine and thiosulfate (PTS-CDs), resulting in a more rigid spherical shape of the CD particles. The neat CDs, US-CDs, and PTS-CDs synthesized at 220 °C for 6 h were 10.3 nm, 7.3 nm, and 2.5 nm in average diameter, respectively. No lattice fringe was observed for neat CDs, indicating an amorphous structure with a high I_D_/I_G_ ratio of 2.63 (Figure 1F). Nevertheless, the carbon lattice fringes were mostly found in US-CDs and PTS-CDs at the synthesis conditions (220 °C, 6 h), as illustrated in Figure S1. The HRTEM images revealed the strip-like structure of US-CDs and PTS-CDs with lattice spacings of 0.24 and 0.21 nm, respectively, which corresponded to the (100) diffraction facet of graphitic carbon, which has lattice spacing between 0.21 and 0.24 nm [19]. The HRTEM image analysis, thus, implies that the PTS-CDs and US-CDs possessed highly graphitic carbon structures in a respective degree according to the Raman spectroscopic analysis with I_D_/I_G_ of 0.61 and 1.52, respectively (Figure 1F). This information indicates that co-doped nitrogen and sulfur CDs (PTS-CDs and US-CDs) were composed of a graphitic sp^2^ carbon atom and sp^3^ carbon defects [20] in different quantities, presented by an integrated peak area, as shown in Figure S2.

**Figure 2:**
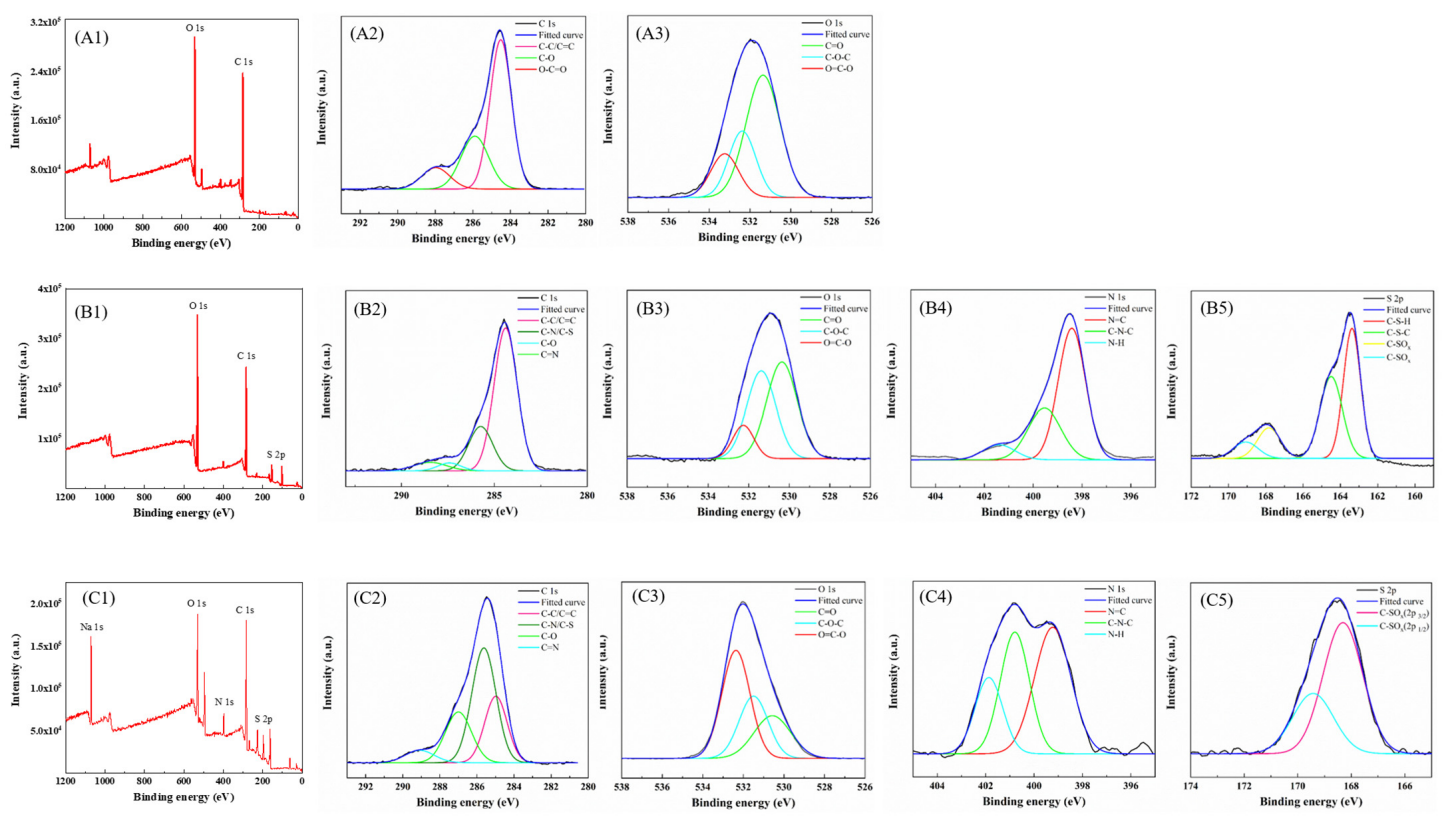
High resolution XPS spectra of (**A**) CDs, (**B**) US-CDs, and (**C**) PTS-CDs for (1) Survey, (2) C1s, (3) O1s, (4) N1s, and (5) S2p.

**Figure 3:**
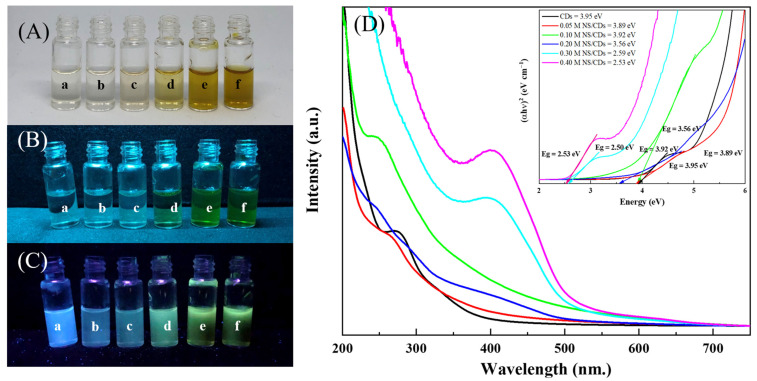
Photographs of the CDs and NS/CDs under (**A**) daylight, (**B**) UV irradiation at 254 nm, and (**C**) UV irradiation at 366 nm for the sample solutions of (a) CDs, (b) 0.05 M NS/CDs, (c) 0.10 M NS/CDs, (d) 0.20 M NS/CDs, (e) 0.30 M NS/CDs, and (f) 0.40 M NS/CDs. (**D**) The UV-visible absorbance of the CDs, 0.05 M NS/CDs, 0.10 M NS/CDs, 0.20 M NS/CDs, 0.30 M NS/CDs, and 0.40 M NS/CDs at the scanned wavelengths from 200 to 700 nm.

The surface state of CDs, US-CDs, and PTS-CDs was identified using an XPS technique, as shown in Figure 2. There were two typical peaks for neat CDs representing O1s and C1s at 532 and 400 eV, respectively (Figure 2A), while nitrogen-and-sulfur-doped CDs (US-CDs and PTS-CDs) consisted of common four XPS peaks at 532, 400, 286, and 164 eV, representing the O1s, N1s, C1s, and S2p shown in Figure 2B and 2C, respectively. In detail, the highly resolved XPS spectrum of C1s of CDs (Figure 2A) prominently exhibited three peaks of C1s states in O-C=O, C-O, and C-C/C=C at binding energies of 288.7, 286.8, and 284.6 eV, respectively, as illustrated in Figure 2(A2). In the case of N-and-S-passivated CDs (US-CDs and PTS-CDs), four deconvoluted C1s peaks were observed. US-CDs and PTS-CDs consisted of four C1s peaks of aforementioned 286.8 eV (C-O) and 284.6 eV (C-C/C=C), including resolved peaks at 285.7 eV assigned to C-N/C-S states and the peak at 288.7 eV attributed to C=N on the surface state, as illustrated in Figure 2B2,C2, respectively. The O1s spectrum (Figure 2C) of all CD, US-CD, and PTS-CD samples can be separated into three peaks, unveiling three oxygen states of C=O at 531.0 eV, C–O–C at 531.7 eV, and O=C–O at 532.8 eV. The highly resolved spectrum of the N1s of US-CDs and PTS-CDs showed the N1s XPS spectra corresponding to N–H, C–N–C, and N=C at binding energies of 401.3, 399.9, and 398.7 eV, respectively (Figure 3B4,C4). The deconvoluted S2p spectra of US-CDs and PTS-CDs (Figure 2B5,C5) display the presence of C–SO_x_ (x = 2–4) species, of which the binding energies were between 168.7–169.85 eV [21,22]. As presented in Figure 2C5, the thiosulfate (S_2_O_3_^2−^) dopant gave a typical sharp spectrum of S2p at 163–164.7 eV different from the sulfate (SO_4_^2−^) dopant, which gave a solely typical S2p peak at 168–169.5 eV, resulting in emerging C-S-H at 163.5 eV and C-S-C at 164.7 eV [23] in PTS-CDs.

In agreement, the XPS spectra for the N1s of US-CDs using urea dopant shown in Figure 2B4 were composed of three peaks at 398.7, 399.9, and 401.3 eV presenting the existence of pyridic nitrogen, pyrrolic nitrogen, and amine groups bound to carbon [24], respectively. In the case of PTS-CDs synthesized by the *p*-phenylenediamine dopant, the deconvoluted N1s XPS spectrum was highly intense for pyridic nitrogen at a 398.7 eV binding energy. Substantially lower intensities of pyrrolic nitrogen attributed to the peak at 399.9 eV and amine groups bound to carbon assigned to the peak at 401.3 eV were detected in carbon materials. The results confirmed that nitrogen atoms were incorporated in the core graphitic carbon of PTS-CDs, indicating that the PTS passivation approach could control the core structure of thermolytically derived CDs. An incorporated sulfur atom that was confirmed by the C-S-C state of S2p peak additionally revealed the induced heteroatom CD synthesis in PTS-CD samples resulting in the enhancement of energy trapped on the surface and, thus, boosted its fluorescent properties dramatically compared with other carbon materials (CDs and US-CDs).

Apart from that, analysis of the relative concentration of each chemical bond from XPS measurement is listed in Table S1. Compared with other bonds of C1s spectra, the highest content of C-C/C=C bond was found at 51.99% for CDs, suggesting the predominant existence of sp^2^/sp^3^ C–C of graphene-like structures for the carbon core of CDs [25]. Nevertheless, N-and-S-doped CDs contained the largest content of C-N/C-S bonds at 46.56% for US-CDs, indicating the two heteroatoms of N and S combined with the C elemental structure in single bonds. Interestingly, in the case of the N1s spectrum of PTS-CDs, the fraction of C-N-C bonds was found at only 13.03%, but predominant pyridic nitrogen possessing an N=C bond was detected at the highest quantity of 47.46%. This was in good accordance with the aforementioned discussion of the heteroatom of pyridic C=N that dramatically enhanced the PL performance of PTS-CDs compared with other CD types. It was reported that pyrrolic N on the CD edge was an imperative configuration of surface defects acting as a photoluminescence center [26]. From O1s spectral data, O=C was primarily found on the surface of CDs and PTS-CDs, revealing that the O elements were favorably possessed in the form of carboxyl groups. The C-S 2p1/2 and C-S 2p3/2 in C-SO_x_-H were mostly found in US-CDs at 67.8 and 32.2%, respectively, which was in good accordance with the C1s data. On the other hand, C-S-C (35.5%) and C-SH (43.2%) were found in relatively high amounts for PTS-CDs.

### 2.2. Effect of Different Concentrations of Nitrogen and Sulfur Passivating Agents on CD Characteristics

The CDs and NS/CDs were synthesized via a one-step hydrothermal method from EFBs in ultrapure water at 220 °C for 6 h. As shown in Figure 3A, the aqueous solutions of the (a) CDs and (b) 0.05 M NS/CDs were colorless, whereas, 0.10, 0.20, 0.30, and 0.40 M NS/CDs (Figure 3A(c–f)) were light brown in daylight. The green fluorescence was found under 254 nm (Figure 3B), and the blue-to-green fluorescence was detected under 366 nm excitation wavelength and varied from undoped CDs to CDs doped with higher concentrations of N and S (Figure 3C).

The composition of the CD cores varied from amorphous to graphitic depending on the nitrogen (*p*-phenylenediamine) and sulfur (sodium thiosulfate) concentrations for NS/CD synthesis. Figure 3D demonstrates that the concentrations at 0.30 and 0.40 M NS/CDs gave NS/CDs with a graphitic carbon core structure. It was reported that significant graphitization occurred either at temperatures greater than 300 °C or, in this case, high concentrations of passivating agents, whilst those below resulted in amorphous particles [27], unless sp^2^/sp-hybridized carbon was employed in the precursor [28]. As illustrated in Figure 3D, the light absorption of 0.30 M NS/CDs and 0.40 M NS/CDs exhibited the existence of π–π * (C=C) and n–π * (C=O) transitions in the carbon core and on the surface of the particles [29]. A graphitic carbon core of CDs, therefore, provided a great influence on their light-harvesting capability. Nonetheless, the fluorescence emission required evaluation with QY, which is the ratio of light emission to the absorption performance of the material.

In addition, it can be seen that there was an absorption peak at around 265 nm observed from neat CDs, 0.05 M NS/CDs, and 0.10 M NS/CDs, which were the samples with small amounts of N and S dopants (Figure 3D). The peak at 240–265 nm (less than 300 nm) was presumably assigned to the π →π * transition of aromatic sp^2^ domains [30] of the conjugated C=C bonds concomitant with a carbon core (carbogenic core) [31]. The UV absorption band at wavelengths over 300 nm was attributed to the existence of n-π * transitions from C=O bonds or other functional groups, such as NH_2_ and SO_3_H [32,33], which in this work were derived from amine and thiosulfate groups, respectively. At high concentrations of N and S dopants (0.30 M NS/CDs and 0.40 M NS/CDs), a prominent UV-vis absorption peak at 400 nm was found. It was reported that the UV-vis absorbance peak at longer wavelengths of 350–400 nm is possibly from the trapping of excited state energy of high-density nitrogen and sulfur at the passivated surface [34].

**Figure 4:**
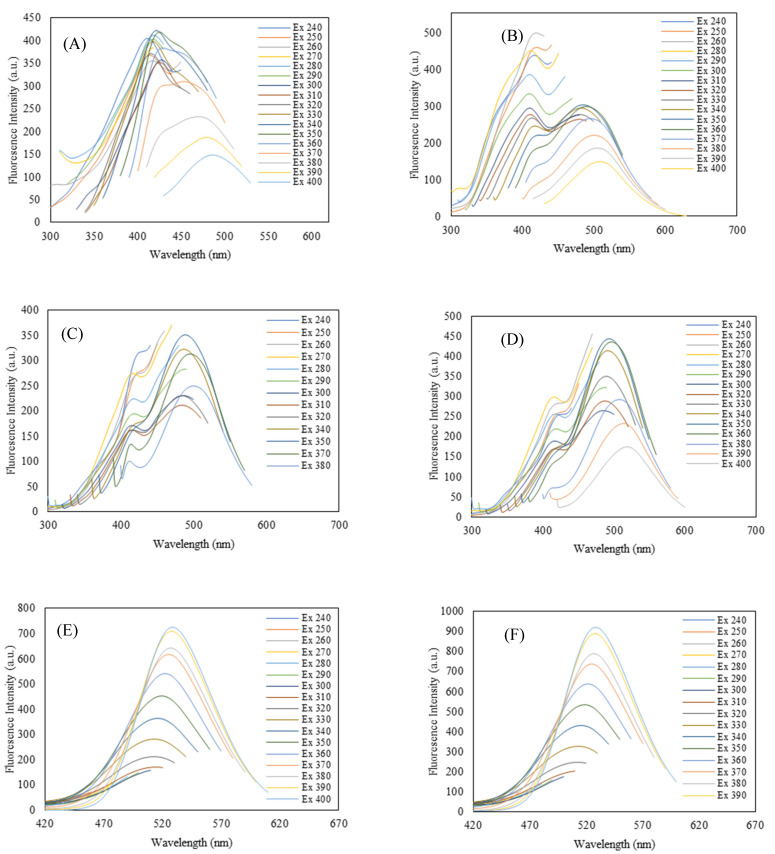
The fluorescence emission spectra of CDs and NS/CDs at various excitation wavelengths from 240 to 400 nm: (**A**) CDs, (**B**) 0.05 M NS/CDs, (**C**) 0.10 M NS/CDs, (**D**) 0.20 M NS/CDs, (**E**) 0.30 M NS/CDs, and (**F**) 0.40 M NS/CDs.

Analyses of the fluorescence properties of the synthesized CDs and NS/CDs at different concentrations of sodium thiosulfate and *p*-phenylenediamine were performed, as illustrated in Figure 4. The highest fluorescence intensity caused by the largest number of particles being excited at that wavelength was observed at excitation wavelengths of 340, 260, 350, 350, 400, and 400 nm for CDs, 0.05 M NS/CDs, 0.10 M NS/CDs, 0.20 M NS/CDs, 0.30 M NS/CDs, and 0.40 M NS/CDs, respectively. When increasing excitation wavelength, the maximum fluorescence emission peak of neat CDs and 0.05 M NS/CDs slightly shifted from a shorter to a longer wavelength, from 400 to 480 nm, and the maximum emission at λ_Em_ = 425 was observed at λ_Ex_ = 330–340 nm excitation wavelength, as shown in Figure 4A,B. For 0.10 M NS/CDs and 0.20 M NS/CDs (Figure 4C,D), the maximum emission wavelength was found at λ_Em_ = 500 at λ_Ex_ = 350. In the case of 0.30 M NS/CDs and 0.40 M NS/CDs, as shown in Figure 4E,F, a substantial shift of the maximum emission peak to λ_Em_ = 530 was detected for both samples at λ_Ex_ = 360. This obvious red-shift phenomenon caused by N-and-S-doping confirmed that either the surface passivation of N and S significantly enhanced the size of the nanoparticles, or the heteroatoms of N and S in the carbon structure apparently reduced the optical bandgap of the CDs when doping with sodium thiosulfate and *p*-phenylenediamine to 0.30 and 0.40 M (0.30 M NS/CDs and 0.40 M NS/CDs). The results were concomitant with a previous report with intense effort to explore the inherent mechanisms controlling the wavelength red-shift of PL emissions within CDs [35]. It has been revealed that red-shifting photoluminescent emission of CDs occurred due to the role of graphitic N, −C−O, −C−N, and −COOH groups on their surface state [36,37,38]. Another report confirmed the green-blue toward green fluorescence emission of prepared SN-CDs derived from doped S atoms relative to the blue emission of N-CDs from doped N atoms [20]. However, due to complications in crystal structure, composition, and surface functionalization of the CDs, only a few studies could evidently identify the PL origin of CDs. Therefore, further thorough examination and demonstration of the relationship between the PL properties of the CDs and the excitation state are essential [39,40].

**Figure 5:**
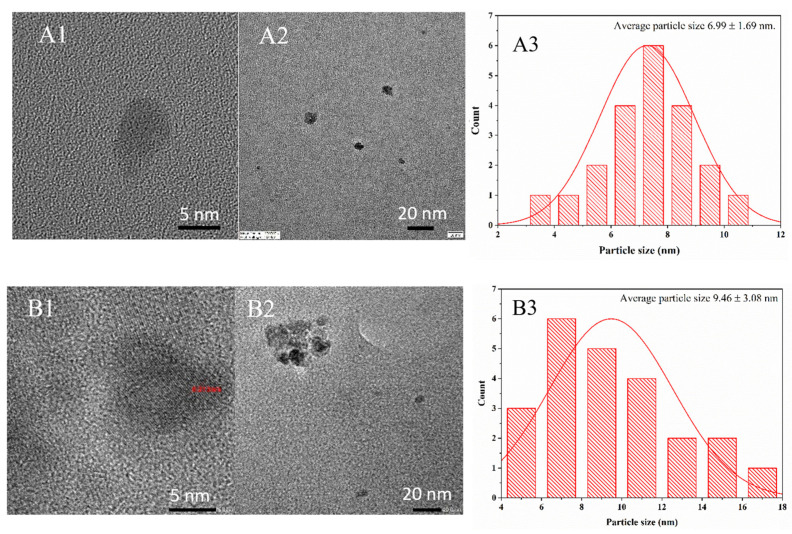

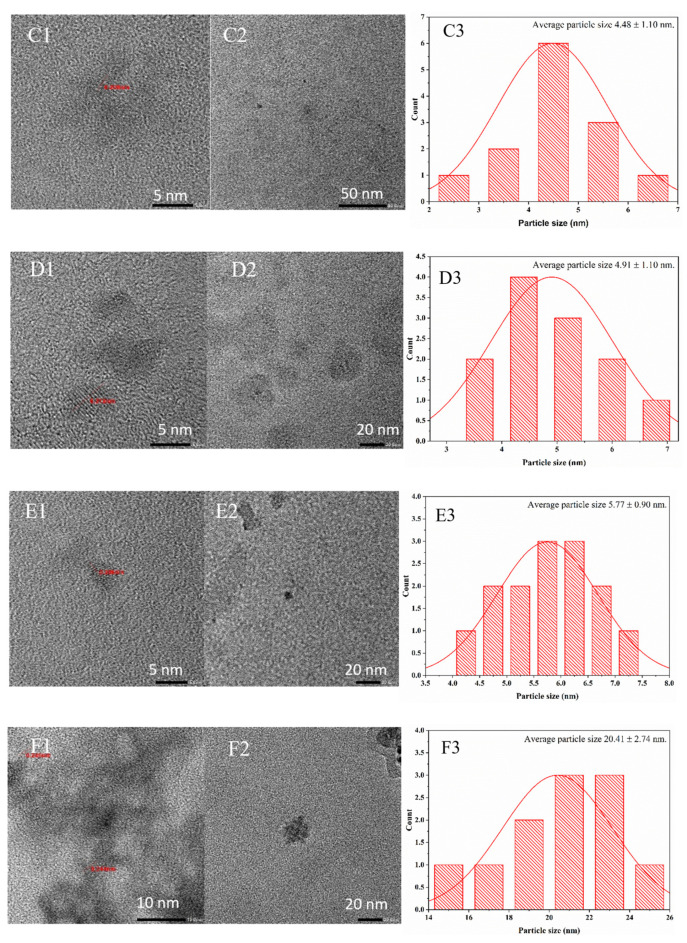
HRTEM images and the size distribution of NS/CDs: (**A**) CDs, (**B**) 0.05 M NS/CDs, (**C**) 0.10 M NS/CDs, (**D**) 0.20 M NS/CDs, (**E**) 0.30 M NS/CDs, and (**F**) 0.40 M NS/CDs. The TEM images: ×100,000 (**left**) and ×150,000 (**middle**) magnification at 200 kV, and the size distribution (**right**).

Apart from that, the emission intensity increased with the increase of N- and S-doping agent concentration, which directly influenced an increase in the QY of NS/CDs, as demonstrated in Table 1. The highest QY of 114% relative to quinine sulfate as the reference (54% QY) was obtained from the 0.40 M NS/CD sample. From Figure 4A,B for neat CDs and 0.05 M NS/CDs, fluorescent intensities were observed as excitation-dependent phenomena that were possibly due to the diverse emissive trap sites on the surface of the NS/CDs [41]. Nevertheless, excitation-independent phenomena occurred for higher concentrations of N and S dopants of 0.1, 0.20, 0.30, and 0.40 M NS/CDs, as depicted in Figure 4C,F. This excitation-dependent fluorescent behavior of NS/CDs was ascribed to either a high density of CD surface state resulting from the synergy effect of the doped nitrogen and sulfur atoms [24] or a highly organized NS/CD surface [42,43]. Compared with virgin undoped CDs, which contained a high density of oxygen atoms on the surface state, N and S co-doped CDs exhibited narrower fluorescence emission spectra. Typically, the effect of different particle sizes reflects excitation-dependent PL behaviors of CDs regarding the distribution of different surface states [44]. The HRTEM images of the CDs and all the NS/CDs shown in Figure 5 indicate that the sizes of all the samples were uniform in the range of 4.48–9.46 nm except for the 0.40 M NS/CDs, which had a diameter of 20.41 nm. Therefore, the excitation-dependent PL properties of the NS/CDs depended on the surface states rather than the morphology. The results suggested that the surface states of the NS/CDs should be rather uniform when increasing the N and S concentrations, leading to provide excitation-dependent behavior with enhanced PL intensity and QY of NS/CDs. The elemental analysis of the surface state of the CDs and NS/CDs was confirmed by XPS data (Table S2).

The optical band gap of CDs and NS/CDs was evaluated using a Tauc plot of the UV-vis absorbance and photon energy with the method of Wood and Tauc [45]. From the UV–vis absorption spectra of CDs, 0.05 M NS/CDs, 0.10 M NS/CDs, 0.20 M NS/CDs, 0.30 M NS/CDs, and 0.40 M NS/CDs, the estimated direct optical band gap energies (E_g_) were 2.54, 3.61, 2.80, 0.73, 0.58, and 0.43 eV, respectively, as shown in Figure 3D and Table 1. When co-doping with nitrogen and sulfur, from 0.05 M to 0.20 M of *p*-phenylenediamine and sodium thiosulfate, the optical band gap of the NS/CDs was reduced in a sequential degree that corresponded to the change from blue- to green-emitting NS/CDs, the so called red-shift phenomenon, under UV excitation at λ_Ex_ = 366 nm (Figure 3C). The reduction of the optical band gap was apparently because of an increase in size [46]. Additionally, highly associated N and S atoms into carbon structure or highly uniform N and S on the surface state were able to manipulate the optical band gap [29]. However, from HRTEM image analysis, the sizes of all the CDs and NS/CDs were uniform between 4 to 9 nm (Figure 5), except for 0.40 M NS/CDs; thus, the surface state played a crucial role in the size of the quantum confinement, which means that N and S co-doping substantially influenced the tunable optical band gap and photonic properties of the NS/CDs. A study revealed that the optical band gap decreased as the structure of N-CDs was transformed from intrinsic C to pyridine N and NH_2_, respectively [47]. Therefore, it could be presumed that N and S co-doping introduced the N π * and S π * orbitals between the C π * and C π orbitals, which could reduce the bandgap energy. This structural change likewise provided ability of NS/CDs to adjust their optical and electrical properties, enhance their chemical reactivity, and avoid self-quenching for further applications [48]. An obvious decrease in the optical band gap in 0.40 M NS/CDs relative to 0.10 M–0.30 M NS/CDs was primarily signified by an increase in the size of the range to 20.4 nm, as illustrated in Figure 5F.

**Figure 6:**
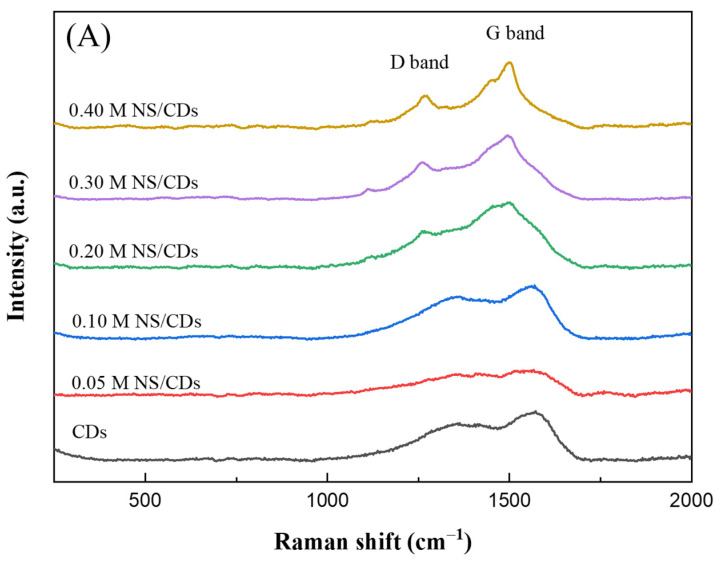

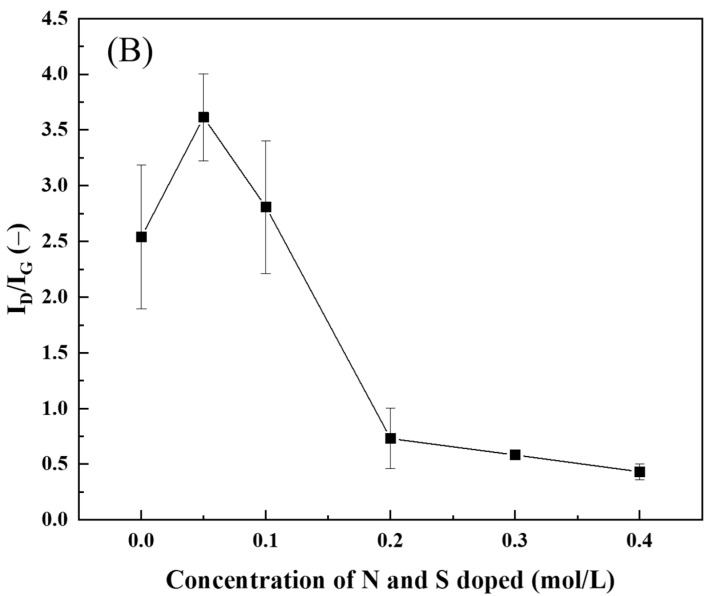
(**A**) Raman spectra and (**B**) I_D_/I_G_ ratio of the CDs, 0.05 M NS/CDs, 0.10 M NS/CDs, 0.20 M NS/CDs, 0.30 M NS/CDs, and 0.40 M NS/CDs.

The HRTEM image analysis in Figure 5 shows that the CDs and all the NS/CDs had a narrow size distribution between 4 nm and 20 nm, especially the 0.20 M NS/CDs and 0.30 M NS/CDs, which gave the smallest size in the range of 4.48 to 4.91 nm (Figure 5C,D). A similar trend in the hydrodynamic diameter of all the CD and NS/CD samples is demonstrated in Table S2. The HRTEM image with greater magnification showed that all the NS/CDs had a lattice spacing (d-spacing) of approximately 0.2–0.3 nm, which is in good agreement with the lattice planes (100) of graphitic carbon, as illustrated in Figure 5B1,F1), while the HRTEM image of neat CDs shows their amorphous structure of carbon core (Figure 5A1). The crystallinity and amorphous morphology of the carbon structures for CDs and NS/CDs was also identified by Raman spectroscopy, demonstrated in Figure 6.

**Scheme 1:**
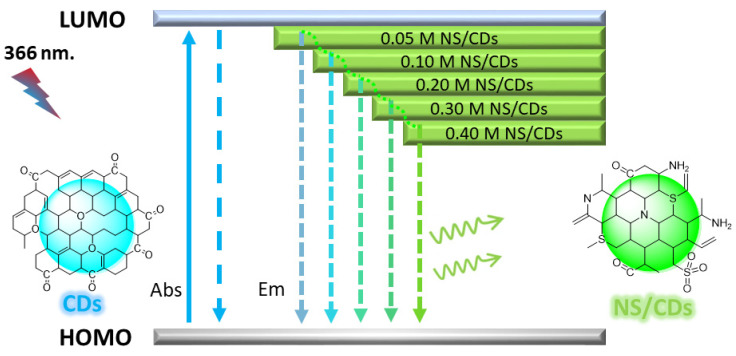
Photonic mechanism of tunable band gap reduction of CDs and NS/CDs by doping with different concentrations of *p*-phenylenediamine (N) and thiosulfate (S).

Scheme 1 presents a photonic model mechanism of CDs and NS/CDs and explains their photoluminescent process. Several hypotheses have stated that surface energy traps and the electronic conjugated structures of CDs provide their photoluminescence [44,49]. Undoped CDs contained the greatest oxygen content on the surface state, as well as a higher ratio of amorphous-to-crystalline carbon structure and, thus, influenced the widest optical band gap and yielded a blue-emitting fluorescence phenomenon. An enhanced N and S concentration in doped CDs led to oxygen reduction with N and S replacement and provided a more homogeneous surface state, resulting in energy level reduction and, therefore, a red-shift of both the excitation and emission band [50]. Moreover, there was evidence confirming that the high QY was because the major state of N dopant resulted from an optimal balance between pyridic N and the amino N on the CD surface; therefore, the electrons and holes were effectively formed as a compound in the forest energy resonance [51]. The presence of an N and S heteroatom from the surface defect passivation on the CD surface was reported to act as excitation energy traps and influence an increase in the photoluminescence of CDs [22]. On the other hand, the sulfur atoms in CDs played a significant role as nanocatalysts for an oxidation-reduction reaction [52], which announced a more passivated CD surface and promoted a boost in photoluminescence.

In addition, the zeta potentials (ζ) of the synthesized CDs, 0.05 M NS/CDs, 0.10 M NS/CDs, 0.20 M NS/CDs, 0.30 M NS/CDs, and 0.40 M NS/CDs were −25.5, −35.5, −46.9, −46.5, −61.9, and −33.4 mV, respectively, as demonstrated in Table 1. The highly negative surface potential obtained could be attributed to the deprotonation of –COOH, –OH, or –NH_2_ on the surface of NS/CDs. It was reported that negative zeta potential was because of the dense electron cloud that concentrates on the surface of CDs [53]. For CDs having a great zeta potential, both negative and positive values are considered stable electrostatically [54], while those having low zeta potentials exhibit weak physical stability and are prone to coagulate or aggregate over a short time period. In the present study, all the NS/CDs were highly dispersed nanoparticles with great stability since they had zeta potentials less than −30 mV or larger than +30 mV [55]. Highly negative zeta potentials beyond −30 mV further demonstrated an effective binding with cations such as heavy metal ions [53], amino groups, and positive charged molecules.

In Raman spectroscopic analysis, the D band at 1385 cm^−1^ and G band at 1575 cm^−1^ are typically used to identify the characteristics of low-dimensional carbon materials such as graphene, graphite, carbon nanotubes, etc. A strong G band peak belongs to highly crystalline carbon structures or graphitic carbon and is identified by sp^2^-hybridized carbon [56]. Notably, the D band is usually observed when the crystalline carbon structure contains defects, impurities [57], or sp^3^ hybridization of the carbon structure [58,59]. As shown in Figure 6A, the G band peak from Raman spectroscopy at 1575 cm^−1^ of 0.20 M NS/CDs, 0.30 M NS/CDs, and 0.40 M NS/CDs was markedly detected, while the presence of the G band in prepared intrinsic CDs, 0.05 M NS/CDs, and 0.10 M NS/CDs was not clear. In the present study, a broad peak of the D band was significantly attributed to a degree of disorder from aromatic conjugated sp^2^ six-membered carbon rings [60]. An increase in the D band and G band width was, moreover, assigned to oxygen-containing groups, including carboxyl, carbonyl, and hydroxyl groups that were introduced onto the basal plane and to the edge of the carbon structure [61]. As demonstrated in Figure 6B, the ratio of disorder of the D band to the crystalline G band (I_D_/I_G_) of high concentrations of N and S dopants in the hydrothermal synthesis ranged between 0.43 and 0.73, similar to that of few-layer graphene quantum dots (1–3 layers) with I_D_/I_G_ of 0.9 [62,63]. The higher crystallinity of carbon or graphitic carbon indicated by a higher content of sp^2^ hybridization was similar to graphene quantum dots (I_D_/I_G_ = 0.5) [64]. The highly graphitic carbon with a high sp^2^/sp^3^ ratio was identified by XPS measurement.

From the highly resolved XPS spectra shown in Figure S3, the C1s deconvoluted peak exhibited the highest content of C-C/C = C in CDs and NS/CDs; the second highest peaks were C=O/C=N and C-O/C-N in a respective degree. An increased N and S concentration seemingly enhanced the C=N pyridic bond structure. In the case of O1s, O=C-O was primarily found in CDs and an increase in N and S concentration increased C=O bonds for 0.05 M to 0.30 M NS/CDs, while 0.40 M NS/CDs contained C-O-C at the highest amount, indicating an oxygen heteroatom was incorporated in the C configuration at a high concentration of N and S dopant. At low concentrations of N and S dopant, C-N-C was found, while the C=N pyridic bond was enhanced at high concentrations of *p*-phenylenediamine (N) for 0.30 M and 0.40 M NS/CDs. In accordance, low concentrations of thiosulfate showed that C-S 2p1/2 and C-S 2p3/2 in the C-SO_x_-H bond were mostly found in 0.05 M to 0.30 M NS/CDs, which was in good agreement with C1s data. On the other hand, C-S-C and C-SH bonds were obviously found in comparatively high amounts for 0.40 M NS/CDs. The consequences were in good agreement with other characterization results, showing extremely greater QY among all samples (Table 1).

Based on our results, we found that CDs with N and S co-doped (NS/CDs) made more narrow bandgap energy than pristine CDs (Figure 3D). Moreover, NS/CDs gave the exclusive properties of a narrow emission wavelength (Figure 4C,F), tunable bandgap, high stability based on zeta potential, and superior fluorescent QY. Regarding its strong graphitic property, high QY, and great stability in aqueous solutions without any chromophore formation (which causes high UV absorption), the 0.20 M NS/CD was selected for further study on fluorescent metal ion sensing application.

### 2.3. Study on Metal Detection by NS/CDs Using Turn-Off Fluorescent Assay

**Figure 7:**
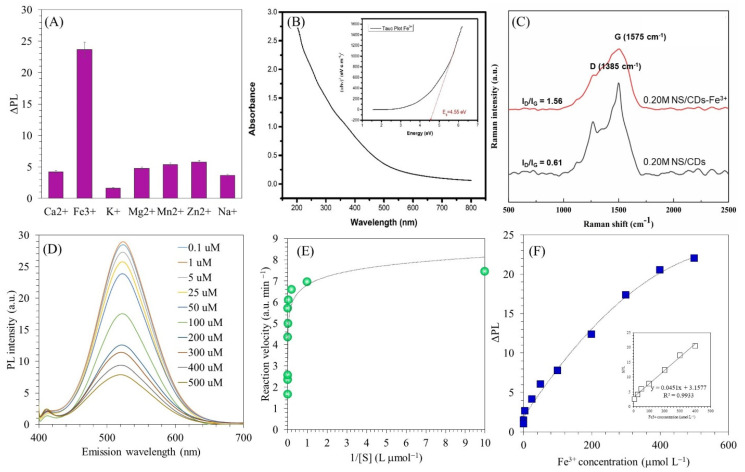
(**A**) Quenching value (ΔPL) of maximum PL emission intensity of 0.20 M NS/CDs at 10 min after binding with various metal ions at 250 μmol L^−1^; (**B**) UV-vis spectroscopy of Fe^3+^ bound to 0.20 M NS/CDs (inset is Tauc plot of optical band gap); (**C**,**D**) monitoring of fluorescence quenching of 0.20 M NS/CDs during reaction with various concentrations of Fe^3+^ at room temperature (25 °C) from 0 to 10 min; (**E**) steady-state kinetic assay for reactions of 0.20 M NS/CDs and Fe^3+^ at room temperature (25 °C); and (**F**) a plot of ΔPL versus reciprocal of Fe^3+^ concentration (inset is a least square linear regression of ΔPL and Fe^3+^ concentration when the reaction time was 10 min).

In an environment of deionized water at pH 5.5, 0.20 M NS/CDs exhibited the strongest fluorescence emission of 520 nm at an excitation wavelength of 380 nm. Therefore, 0.20 M NS/CDs were employed as a fluorescence probe to detect metal ions at low concentrations in deionized water. As shown in Figure 7, Fe^3+^ was successfully detected with the highest efficacy and sensitivity compared to other metal ions, including Ca^2+^, K^+^, Mg^2+^, Mn^2+^, Zn^2+^, and Na^+^. The metal ions employed in this experiment are mostly found in human biofluids such as tears [65] and sweat [66]. The surface state of 0.20 M NS/CDs enriched with COOH, C-OH, C-O, and C=O functional groups that were indicated by XPS analysis and the negative charges of 0.20 M NS/CDs expressed by zeta potential evidently facilitated highly sensitive and effective binding of 0.20 M NS/CDs toward Fe^3+^ ions. Fe^3+^ ions were also found to be effectively absorbed on the CD surface and coordinated with hydroxyl groups on edges of CDs [67]. From this high affinity of Fe^3+^ and hydroxyl group coordination interaction on the CD surface, electrons generated from the excited state of NS/CDs apparently transferred to the Fe^3+^ unfilled orbitals, which lead to nonradiative electron–hole recombination resulting in the highest degree of CD fluorescence quenching [22] compared with other metal ions, as shown in Figure 7A.

Interestingly, the optical band gap of 0.20 M NS/CDs and Fe^3+^ was enhanced to 4.55 eV after Fe^3+^ absorption on the surface of CDs, which was much greater than the optical band gap of freshly prepared 0.20 M NS/CDs at 3.56 eV (Figure 7B). The reason was possibly because of the change in microstructure of nanomaterial either from highly crystalline to, most likely, amorphous or from small size to a larger size. This was confirmed by a Raman spectroscopic analysis of Fe^3+^ absorbed on 0.20 M NS/CDs (Figure 7C), which substantially altered the graphitic structure of 0.20 M NS/CDs by increasing the I_D_/I_G_ ratio from 0.61 to 1.56, indicating more defects of the graphitic structure of NS/CDs corresponding to the sp^3^ vibrations of disordered graphite layers. An increase in Fe^3+^ concentration provided greater PL quenching to a corresponding degree, as illustrated in Figure 7D. Figure 7E demonstrates a plot of the reaction velocity (ΔPL/Δt) versus the reciprocal of Fe^3+^ concentration for a pseudo-steady-state kinetic assay study, and a plot of ΔPL and Fe^3+^ concentration with an inset of a least square linear regression of ΔPL and Fe^3+^ concentration at the reaction time of 10 min demonstrated the best fit of the linear equation ΔPL = 0.0451 [Fe^3+^] + 3.1577 with R^2^ = 0.9933 when [Fe^3+^] is the concentration in μmol L^−1^ (Figure 7F). The linear range of Fe^3+^ sensing in this study was from 5 to 400 μmol L^−1^, which was a very wide range with a relatively low limit of detection (LOD) at 0.1 μmol L^−1^ or 100 nmol L^−1^. The findings showed that our 0.20 M NS/CD material is a promising Fe^3+^ sensing agent giving a wide range of detection with a very low LOD amongst other agents recently reported, as demonstrated in Table 2.

## 3. Materials and Methods

The palm empty fruit bunches were supplied from a palm oil mill, Chumporn province, Thailand. The chemicals, namely urea and sodium thiosulfate, were purchased from Ajax Finechem (New South Wales, Australia). Sulfuric acid at 98% was purchased from RCI Labscan (Bangkok, Thailand). *P*-phenylenediamine was purchased from Alfa Aesar (Haverhill, MA, USA). Other metals, including potassium chloride (KCl or K^+^), manganese chloride (MnCl_2_ or Mn^2+^) and iron(III) chloride (FeCl_3_ or Fe^3+^), were purchased from Ajax Finechem (New South Wales, Australia). Sodium chloride (NaCl or Na^+^) and calcium chloride (CaCl_2_ or Ca^+^) were purchased from Alpha Chemika (Maharashtra, India). Magnesium chloride (MgCl_2_ or Mg^2+^) was purchased from Riedel de Haen (Seelze, Germany). Zinc chloride (ZnCl_2_ or Zn^2+^) was purchased from QReC (Kuala Lumpur, Malaysia). All the chemicals and metals were analytical grade and were utilized as received without modification. Ultrapure water was used for the preparation of the carbon dots, reagent solutions, and stock solutions of metal ions. A dialysis membrane (molecular weight cut-off of 1000 Da) was purchased from Spectra/por (Spectrum, Cole-Palmer, IL, USA), and a nylon syringe filter (0.22 μm) was supplied from Filtrex (Milano, Italy).

### 3.1. Hydrothermal Synthesis of Carbon Dots from EFBs and Effect of Different Dopants on CD Properties

**Figure 8:**
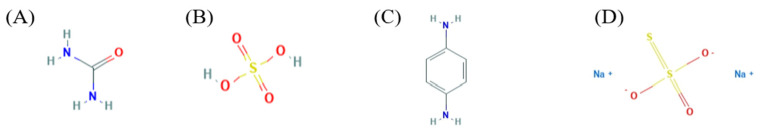
Nitrogen and sulfur passivating agents for NS/CD synthesis from EFBs using hydrothermal method: (**A**) urea (U), (**B**) sulfuric acid (S), (**C**) *p*-phenylenediamine (P), and (**D**) sodium thiosulfate (TS).

The EFBs were washed with deionized water, sun-dried for 1–2 days, and dried in the oven at 100 °C for 24 h to obtain a moisture content of 3–4%. Then, the EFB fiber was chopped into 1–2-inch pieces, milled, and sieved through +50/−200 mesh. The CDs were synthesized using a hydrothermal method in a Teflon-lined hydrothermal stainless steel autoclave. The milled EFB fiber (5 g) was mixed with 60 mL of ultrapure water (solid:liquid ratio of 1:12) and underwent a hydrothermal reaction at 220 °C for 6 h. For the case of surface passivation, nitrogen-doped CDs were prepared by mixing milled EFB fiber (5 g) and 60 mL of 0.1 mol L^−1^ urea (U-CDs) or 0.1 mol L^−1^ of *p*-phenylenediamine (P-CDs) under hydrothermal conditions at 220 °C for 6 h. Nitrogen- and sulfur- (N- and S-) passivated CDs were prepared by mixing milled EFB fiber (5 g) with 60 mL of a solution mixture of 0.2 mol L^−1^ urea and 0.2 mol L^−1^ sulfuric acid (US-CDs) or 0.2 mol L^−1^ *p*-phenylenediamine and 0.2 mol L^−1^ sodium thiosulfate (PTS-CDs) under hydrothermal conditions at 220 °C for 6 h. The molecular structures of the N and S dopants are shown in Figure 8. After the reaction, the materials were separated from the solution by using a centrifugal separator at a speed of 5000 rpm to separate the cellulose-rich fraction and, subsequently, 10,000 rpm to separate hemicellulose and lignin from the solution for 15 min at 20 °C. Purification of the CDs was carried out using a 0.22 μm syringe-type membrane filter, and the CDs were finally dialyzed using a membrane (1000 Dalton molecular weight cut-off).

### 3.2. Effect of N and S Concentrations on Enhancement of CD Photoluminescent Properties

To enhance and tune CD photoluminescence properties, surface passivation with nitrogen and sulfur atoms was performed. Based on data from previous experiments, nitrogen (N)-doping with *p*-phenylenediamine and sulfur (S)-doping with sodium thiosulfate were conducted in a one-pot hydrothermal synthesis of the CDs. The N and S concentrations were varied from 0.05 to 0.40 mol L^−1^ and named as 0.05 M NS/CDs to 0.40 M NS/CDs, respectively. Finally, brown solutions of the CDs and NS/CDs were separated from the solid residue and purified using centrifugation, microfiltration, and dialysis, as aforementioned. The clear brown solutions of CDs and the NS/CDs obtained were stored at 4 °C for further use and characterization. Dry solid samples of the CDs and NS/CDs for characterization were prepared using freeze-drying.

### 3.3. Characterization of CDs

The fluorescence measurement of the CD and NS/CD solutions was performed using a Model FP-6200 Spectrofluorometer (Perkin Elmer, Kumamoto, Japan) with a scan speed of 500 nm min^−1^ and an excitation and emission slit width of 10 nm. The fluorescence behavior was studied by varying the excitation wavelength in the range of 240–400 nm. The morphological characterizations were evaluated by a JEM-2100 Plus High-resolution Transmission Electron Microscope (HRTEM) (JEOL, Seoul, Republic of Korea) operated at 200 kV. Zeta potential measurement was conducted in triplicate for each sample with a nanoPartica SZ-100 V2 Series (Malvern Panalytical, Cambridge, United Kingdom). The carbon structures of the CDs and NS/CDs were characterized with an XploRA PLUS Raman microscope (Thermo Fisher Scientific, Massachusetts, USA) using a 532 nm laser as the excitation source with a slit of 100 μm in the range of 3700–100 cm^−1^. The surface elemental composition of the samples was investigated using X-ray photoelectron spectroscopy (XPS) with a PHI5000 VersaProbe II (ULVAC-PHI, Kanagawa, Japan) at the SUT-NANOTEC-SLRI Joint Research Facility, Synchrotron Light Research Institute (SLRI, Nakhon Ratchasima, Thailand). A monochromatized Al-Kα X-ray source (hγ = 1486.6 eV) was used to excite the specimens. The survey spectra were recorded with an energy step of 1.000 eV and a pass energy of 117.4 eV; meanwhile, the high-resolution spectra were recorded with an energy step of 0.05 eV and a pass energy of 46.95 eV. A UV-vis double-beam spectrophotometer (UV-1800, Thermo Fisher Scientific, Japan) was used to record the UV-vis absorbance of the synthesized CDs and NS/CDs with a scanning wavelength range between 200 and 800 nm, a scanning speed of 500 nm min^−1^, and a bandwidth slit of 1 nm.

To determine the quantum yield of the CD samples, quinine sulfate (QY = 54%) dissolved in 0.1 M H_2_SO_4_ solution was used as a reference (Ref). The QY of the CDs and NS/CDs in an aqueous solution was calculated using Equation (1):

(1) $${\mathrm{QY}}_{\mathrm{CDs}}={\mathrm{QY}}_{\mathrm{Ref}}\times \frac{I_{\mathrm{CDs}}}{I_{\mathrm{Ref}}}\times \frac{A_{\mathrm{Ref}}}{A_{\mathrm{CDs}}}\times \frac{\eta_{\mathrm{CDs}}^{2}}{\eta_{\mathrm{Ref}}^{2}}$$

where I is the integrated intensity of the fluorescent spectra, A is the UV-vis absorbance, η is the refractive index of the solvent (ultrapure water for CDs and 0.1 M H_2_SO_4_ for quinine sulfate), and QY is the quantum yield (%) [76].

### 3.4. Study on Metal Detection by NS/CDs Using Fluorimetric Sensing

The study on metal ions detection using the highest potential 0.2 M NS/CDs from the previous experiment was performed when the nitrogen (N) dopant was *p*-phenylenediamine and the sulfur (S) dopant was sodium thiosulfate. The fluorescent quenching responses of the 0.20 M NS/CDs toward various metal ion bindings were investigated. First, a stock solution of metal chlorides at 500 μmol L^−1^ was prepared, including Ca^2+^, Fe^3+^, K^+^, Mg^2+^, Mn^2+^, Zn^2+^, and Na^+^. For the kinetic study, 1 mL of 500 μmol L^−1^ metal chloride stock solution was mixed with 0.25 mL ultrapure water and 0.75 mL 0.2 M NS/CDs (0.25 mg mL^−1^ concentration). The final concentration of the reaction mixture contained 250 μmol L^−1^ metal chloride and 100 μg mL^−1^ 0.20 M NS/CDs. Once the metal chloride solution was added, the excitation (λ_Ex_) at 380 nm of 0.20 M NS/CDs yielding a maximum photoluminescence emission intensity at 520 nm (λ_Em_) was recorded from 0 to 10 min for kinetic study. The velocity of the absorption of various metal ions into 0.20 M NS/CDs was calculated. To examine the sensitivity of Fe^3+^ sensing using 0.20 M NS/CDs, the concentration of Fe^3+^ was varied between 0.1 and 500 μmol L^−1^, and the change of PL intensity (ΔPL) was monitored at 10 min at λ_Ex_ = 380 nm. A linear correlation of ΔPL and Fe^3+^ concentration was determined, as well as the limit of detection (LOD) of Fe^3+^ using 0.20 M NS/CDs.

## 4. Conclusions

CDs were successfully synthesized from palm empty fruit bunches through a hydrothermal approach yielding a spherical shape of the carbon core structure with a narrow size distribution and an average diameter ranging from 2.5 to 10.3 nm. Passivation of CDs with *p*-phenylenediamine and thiosulfate provided NS/CDs with outstanding QY with highly selective affinity for Fe^3+^ in deionized water. The decrease in CD size from N and S doping to the optimal concentrations of N and S resulted in an increase in the optical bandgap and PL emissions in the high-energy region. Fluorescence sensing using NS/CDs as a fluorescent nanoprobe could detect Fe^3+^ at very low concentrations with an LOD of 0.1 μmol L^−1^ or 100 nmol L^−1^ through a fluorescence quenching mechanism. The linearity of detection was found in the range of 5 to 400 μmol L^−1^, which is a substantially greater range than recently reported nanoprobes. Taking into account the highly luminescent, cost-effective, highly soluble, and sensitive sensing capabilities of NS/CDs even at low concentrations of Fe^3+^, these make as-prepared NS/CDs an ultimate candidate for prospective applications in sensing other metal ions and biomolecules, such as in-depth pathophysiological insights into different clinical stages of iron-related neurodegeneration.

## Figures and Tables

**Table 1:** Concentration, QY, zeta potential, optical band gap, and ID/IG ratio from Raman spectroscopy of CDs, 0.05 M NS/CDs, 0.10 M NS/CDs, 0.20 M NS/CDs, 0.30 M NS/CDs, and 0.40 M NS/CDs.

Sample	QY (%)	ζ (mV)	E_g_ (eV)	I_D_/I_G_ Ratio
CDs	39%	−25.5	3.95	2.54
0.05 M NS/CDs	24%	−35.5	3.89	3.61
0.10 M NS/CDs	18%	−46.9	3.92	2.80
0.20 M NS/CDs	48%	−46.5	3.56	0.73
0.30 M NS/CDs	75%	−61.9	2.59	0.58
0.40 M NS/CDs	114%	−33.4	2.53	0.43

**Table 2:** Comparison of the performance of the as-prepared 0.20 M NS/CDs with other fluorescent nanoprobes.

Detection Probe	Ions Detected	Detection Limit (μM)	Linear Range (μM)	References
Graphene quantum dots (GQDs)	Fe^3+^	7.22	0–80	[68]
Nitrogen-doped carbon dots (N-CDs)	Fe3^+^	0.96	0–100	[69]
Nitrogen-doped polymer nanodots (N-PNDs)	Fe^3+^	0.10	0–30	[70]
Graphene oxide (GO) nanosheets	Fe^3+^	17.50	14.3–143.2	[71]
Fluorescent carbon nanoparticles (F-CNPs)	Fe^3+^	0.32	0–20	[72]
Phosphorus-doped carbon quantum dots (P-CQDs)	Fe^3+^, Fe^2+^	0.005	0–20	[73]
Nitrogen-doped carbon quantum dots (N-CQDs)	Fe^3+^, Hg^2+^	0.09	1–1945	[5]
Au nanoclusters	Fe^3+^	3.50	5–1280	[74]
Nitrogen-doped carbon quantum dots (NCQDs)	Fe^3+^	4.67	0–50	[75]
Nitrogen-and-sulfur-doped carbon dots (0.20 M NS/CDs)	Fe^3+^	0.10	5–400	This study

## Data Availability

Not applicable.

## Supplementary Materials

The following supporting information can be downloaded at: https://www.mdpi.com/article/10.3390/ijms23095001/s1.

## References

[1] Li S., Li Y., Cao J., Zhu J., Fan L., Li X. (2014). Sulfur-Doped Graphene Quantum Dots as a Novel Fluorescent Probe for Highly Selective and Sensitive Detection of Fe^3+^. Anal. Chem..

[2] Zecca L., Youdim M.B.H., Riederer P., Connor J.R., Crichton R.R. (2004). Iron, brain ageing and neurodegenerative disorders. Nat. Rev. Neurosci..

[3] Liu Y., Nguyen M., Robert A., Meunier B. (2019). Metal Ions in Alzheimer’s Disease: A Key Role or Not?. Acc. Chem. Res..

[4] Ju J., Chen W. (2014). Synthesis of highly fluorescent nitrogen-doped graphene quantum dots for sensitive, label-free detection of Fe (III) in aqueous media. Biosens. Bioelectron..

[5] Sui B., Tang S., Liu T., Kim B., Belfield K.D. (2014). Novel BODIPY-Based Fluorescence Turn-on Sensor for Fe^3+^ and Its Bioimaging Application in Living Cells. ACS Appl. Mater. Interfaces.

[6] Ghaedi M., Shokrollahi A., Kianfar A.H., Mirsadeghi A.S., Pourfarokhi A., Soylak M. (2008). The determination of some heavy metals in food samples by flame atomic absorption spectrometry after their separation-preconcentration on bis salicyl aldehyde, 1,3 propan diimine (BSPDI) loaded on activated carbon. J. Hazard. Mater..

[7] Farzana Akter K., Chen Z., Smith L., Davey D., Naidu R. (2005). Speciation of arsenic in ground water samples: A comparative study of CE-UV, HG-AAS and LC-ICP-MS. Talanta.

[8] Zeng Z., Yu D., He Z., Liu J., Xiao F.-X., Zhang Y., Wang R., Bhattacharyya D., Tan T.T.Y. (2016). Graphene Oxide Quantum Dots Covalently Functionalized PVDF Membrane with Significantly-Enhanced Bactericidal and Antibiofouling Performances. Sci. Rep..

[9] Liu S., Zhang X., Yu Y., Zou G. (2014). A Monochromatic Electrochemiluminescence Sensing Strategy for Dopamine with Dual-Stabilizers-Capped CdSe Quantum Dots as Emitters. Anal. Chem..

[10] Campos B.B., Mutavdžić D., Stanković M., Radotić K., Lázaro-Martínez J.M., Esteves da Silva J.C.G., Contreras-Cáceres R., Pino-González M.S., Rodriguez-Castellón E., Algarra M. (2017). Thermo-responsive microgels based on encapsulated carbon quantum dots. New J. Chem..

[11] Liu Z., Xiao J., Wu X., Lin L., Weng S., Chen M., Cai X., Lin X. (2016). Switch-on fluorescent strategy based on N and S co-doped graphene quantum dots (N-S/GQDs) for monitoring pyrophosphate ions in synovial fluid of arthritis patients. Sens. Actuators B Chem..

[12] Zhang L., Zhang Z.-Y., Liang R.-P., Li Y.-H., Qiu J.-D. (2014). Boron-Doped Graphene Quantum Dots for Selective Glucose Sensing Based on the “Abnormal” Aggregation-Induced Photoluminescence Enhancement. Anal. Chem..

[13] Fedorenko S., Stepanov A., Bochkova O., Kholin K., Dovjenko A., Zairov R., Nizameev I., Gerasimova T., Strelnik I., Voloshina A. (2021). Tailoring of silica nanoarchitecture to optimize Cu_(2−x)_S based image-guided chemodynamic therapy agent. Colloids Surf. A Physicochem. Eng. Asp..

[14] Zairov R.R., Dovzhenko A.P., Sarkanich K.A., Nizameev I.R., Luzhetskiy A.V., Sudakova S.N., Podyachev S.N., Burilov V.A., Vatsouro I.M., Vomiero A. (2021). Single Excited Dual Band Luminescent Hybrid Carbon Dots-Terbium Chelate Nanothermometer. Nanomaterials.

[15] Atabaev T.S. (2018). Doped Carbon Dots for Sensing and Bioimaging Applications: A Minireview. Nanomaterials.

[16] Liu Y., Jiang L., Li B., Fan X., Wang W., Liu P., Xu S., Luo X. (2019). Nitrogen doped carbon dots: Mechanism investigation and their application for label free CA125 analysis. J. Mater. Chem. B.

[17] Guo R., Chen B., Li F., Weng S., Zheng Z., Chen M., Wu W., Lin X., Yang C. (2018). Positive carbon dots with dual roles of nanoquencher and reference signal for the ratiometric fluorescence sensing of DNA. Sens. Actuators B Chem..

[18] Hutton G.A.M., Reuillard B., Martindale B.C.M., Caputo C.A., Lockwood C.W.J., Butt J.N., Reisner E. (2016). Carbon Dots as Versatile Photosensitizers for Solar-Driven Catalysis with Redox Enzymes. J. Am. Chem. Soc..

[19] Dong Y., Zhou N., Lin X., Lin J., Chi Y., Chen G. (2010). Extraction of Electrochemiluminescent Oxidized Carbon Quantum Dots from Activated Carbon. Chem. Mater..

[20] Hu Y., Zhang L., Li X., Liu R., Lin L., Zhao S. (2017). Green Preparation of S and N Co-Doped Carbon Dots from Water Chestnut and Onion as Well as Their Use as an Off–On Fluorescent Probe for the Quantification and Imaging of Coenzyme A. ACS Sustain. Chem. Eng..

[21] Shen J., Zhang T., Cai Y., Chen X., Shang S., Li J. (2017). Highly fluorescent N,S-co-doped carbon dots: Synthesis and multiple applications. New J. Chem..

[22] Xu Q., Pu P., Zhao J., Dong C., Gao C., Chen Y., Chen J., Liu Y., Zhou H. (2014). Preparation of highly photoluminescent sulfur-doped carbon dots for Fe(iii) detection. J. Mater. Chem. A.

[23] Munir A., Haq Tu Qurashi A., Rehman Hu Ul-Hamid A., Hussain I. (2019). Ultrasmall Ni/NiO Nanoclusters on Thiol-Functionalized and Exfoliated Graphene Oxide Nanosheets for Durable Oxygen Evolution Reaction. ACS Appl. Energy Mater..

[24] Martindale B.C.M., Hutton G.A.M., Caputo C.A., Prantl S., Godin R., Durrant J.R., Reisner E. (2017). Enhancing Light Absorption and Charge Transfer Efficiency in Carbon Dots through Graphitization and Core Nitrogen Doping. Angew. Chem. Int. Ed..

[25] Wang Y., Kim S.-H., Feng L. (2015). Highly luminescent N,S-Co-doped carbon dots and their direct use as mercury(II) sensor. Anal. Chim. Acta.

[26] Dong Y., Pang H., Yang H.B., Guo C., Shao J., Chi Y., Li C.M., Yu T. (2013). Carbon-Based Dots Co-doped with Nitrogen and Sulfur for High Quantum Yield and Excitation-Independent Emission. Angew. Chem. Int. Ed..

[27] Cayuela A., Soriano M.L., Carrillo-Carrión C., Valcárcel M. (2016). Semiconductor and carbon-based fluorescent nanodots: The need for consistency. Chem. Commun..

[28] Moon B.J., Oh Y., Shin D.H., Kim S.J., Lee S.H., Kim T.-W., Park M., Bae S. (2016). Facile and Purification-Free Synthesis of Nitrogenated Amphiphilic Graphitic Carbon Dots. Chem. Mater..

[29] Li X., Zhang S., Kulinich S.A., Liu Y., Zeng H. (2014). Engineering surface states of carbon dots to achieve controllable luminescence for solid-luminescent composites and sensitive Be^2+^ detection. Sci. Rep..

[30] Eda G., Lin Y.-Y., Mattevi C., Yamaguchi H., Chen H.-A., Chen I.-S., Chen C.-W., Chhowalla M. (2010). Blue Photoluminescence from Chemically Derived Graphene Oxide. Adv. Mater..

[31] Wibrianto A., Khairunisa S.Q., Sakti S.C.W., Ni’mah Y.L., Purwanto B., Fahmi M.Z. (2021). Comparison of the effects of synthesis methods of B, N, S, and P-doped carbon dots with high photoluminescence properties on HeLa tumor cells. RSC Adv..

[32] Chao D., Chen J., Dong Q., Wu W., Qi D., Dong S. (2020). Ultrastable and ultrasensitive pH-switchable carbon dots with high quantum yield for water quality identification, glucose detection, and two starch-based solid-state fluorescence materials. Nano Res..

[33] Dhenadhayalan N., Lin K.-C., Suresh R., Ramamurthy P. (2016). Unravelling the Multiple Emissive States in Citric-Acid-Derived Carbon Dots. J. Phys. Chem. C.

[34] Anilkumar P., Wang X., Cao L., Sahu S., Liu J.-H., Wang P., Korch K., Tackett Ii K.N., Parenzan A., Sun Y.-P. (2011). Toward quantitatively fluorescent carbon-based “quantum” dots. Nanoscale.

[35] Zhang Q., Wang R., Feng B., Zhong X., Ostrikov K. (2021). Photoluminescence mechanism of carbon dots: Triggering high-color-purity red fluorescence emission through edge amino protonation. Nat. Commun..

[36] Ding H., Yu S.-B., Wei J.-S., Xiong H.-M. (2016). Full-Color Light-Emitting Carbon Dots with a Surface-State-Controlled Luminescence Mechanism. ACS Nano.

[37] Holá K., Sudolská M., Kalytchuk S., Nachtigallová D., Rogach A.L., Otyepka M., Zbořil R. (2017). Graphitic Nitrogen Triggers Red Fluorescence in Carbon Dots. ACS Nano.

[38] Pan L., Sun S., Zhang A., Jiang K., Zhang L., Dong C., Huang Q., Wu A., Lin H. (2015). Truly Fluorescent Excitation-Dependent Carbon Dots and Their Applications in Multicolor Cellular Imaging and Multidimensional Sensing. Adv. Mater..

[39] Antaris A.L., Chen H., Cheng K., Sun Y., Hong G., Qu C., Diao S., Deng Z., Hu X., Zhang B. (2016). A small-molecule dye for NIR-II imaging. Nat. Mater..

[40] Wu Z.L., Liu Z.X., Yuan Y.H. (2017). Carbon dots: Materials, synthesis, properties and approaches to long-wavelength and multicolor emission. J. Mater. Chem. B.

[41] Lu H., Li C., Wang H., Wang X., Xu S. (2019). Biomass-Derived Sulfur, Nitrogen Co-Doped Carbon Dots for Colorimetric and Fluorescent Dual Mode Detection of Silver (I) and Cell Imaging. ACS Omega.

[42] Tao S., Lu S., Geng Y., Zhu S., Redfern S.A.T., Song Y., Feng T., Xu W., Yang B. (2018). Design of Metal-Free Polymer Carbon Dots: A New Class of Room-Temperature Phosphorescent Materials. Angew. Chem. Int. Ed..

[43] Han Z., Zhang H., He L., Pan S., Liu H., Hu X. (2019). One-pot hydrothermal synthesis of nitrogen and sulfur co-doped carbon dots and their application for sensitive detection of curcumin and temperature. Microchem. J..

[44] Sun Y.-P., Zhou B., Lin Y., Wang W., Fernando K.A.S., Pathak P., Meziani M.J., Harruff B.A., Wang X., Wang H. (2006). Quantum-Sized Carbon Dots for Bright and Colorful Photoluminescence. J. Am. Chem. Soc..

[45] Yang J., Chen W., Liu X., Zhang Y., Bai Y. (2017). Hydrothermal synthesis and photoluminescent mechanistic investigation of highly fluorescent nitrogen doped carbon dots from amino acids. Mater. Res. Bull..

[46] Rabouw F.T., de Mello Donega C. (2016). Excited-State Dynamics in Colloidal Semiconductor Nanocrystals. Top. Curr. Chem..

[47] Yu J., Liu C., Yuan K., Lu Z., Cheng Y., Li L., Zhang X., Jin P., Meng F., Liu H. (2018). Luminescence Mechanism of Carbon Dots by Tailoring Functional Groups for Sensing Fe^3+^ Ions. Nanomaterials.

[48] Xu S., Liu Y., Yang H., Zhao K., Li J., Deng A. (2017). Fluorescent nitrogen and sulfur co-doped carbon dots from casein and their applications for sensitive detection of Hg^2+^ and biothiols and cellular imaging. Anal. Chim. Acta.

[49] Wang X., Cao L., Yang S.T., Lu F., Meziani M.J., Tian L., Sun K.W., Bloodgood M.A., Sun Y.P. (2010). Bandgap-like strong fluorescence in functionalized carbon nanoparticles. Angew. Chem. Int. Ed. Engl..

[50] Qu D., Sun Z., Zheng M., Li J., Zhang Y., Zhang G., Zhao H., Liu X., Xie Z. (2015). Three Colors Emission from S,N Co-doped Graphene Quantum Dots for Visible Light H_2_ Production and Bioimaging. Adv. Opt. Mater..

[51] Cui S., Wu Y., Liu Y., Guan Q., Zhang Y., Zhang Y., Luo S., Xu M., Wang J. (2020). Synthesis of carbon dots with a tunable photoluminescence and their applications for the detection of acetone and hydrogen peroxide. Chin. Chem. Lett..

[52] Liang J., Jiao Y., Jaroniec M., Qiao S.Z. (2012). Sulfur and Nitrogen Dual-Doped Mesoporous Graphene Electrocatalyst for Oxygen Reduction with Synergistically Enhanced Performance. Angew. Chem. Int. Ed..

[53] Yahaya Pudza M., Zainal Abidin Z., Abdul Rashid S., Md Yasin F., Noor A.S.M., Issa M.A. (2020). Eco-Friendly Sustainable Fluorescent Carbon Dots for the Adsorption of Heavy Metal Ions in Aqueous Environment. Nanomaterials.

[54] Li W., Wang S., Li Y., Ma C., Huang Z., Wang C., Li J., Chen Z., Liu S. (2017). One-step hydrothermal synthesis of fluorescent nanocrystalline cellulose/carbon dot hydrogels. Carbohydr. Polym..

[55] Clogston J.D., Patri A.K. (2011). Zeta potential measurement. Methods Mol. Biol..

[56] Papaioannou N., Marinovic A., Yoshizawa N., Goode A.E., Fay M., Khlobystov A., Titirici M.-M., Sapelkin A. (2018). Structure and solvents effects on the optical properties of sugar-derived carbon nanodots. Sci. Rep..

[57] Yoshikawa M., Mori Y., Obata H., Maegawa M., Katagiri G., Ishida H., Ishitani A. (1995). Raman scattering from nanometer-sized diamond. Appl. Phys. Lett..

[58] Liu H., Zhang Y., Huang C. (2019). Development of nitrogen and sulfur-doped carbon dots for cellular imaging. J. Pharm. Anal..

[59] Reckmeier C.J., Schneider J., Susha A.S., Rogach A.L. (2016). Luminescent colloidal carbon dots: Optical properties and effects of doping [Invited]. Opt. Express.

[60] Castiglioni C., Negri F., Rigolio M., Zerbi G. (2001). Raman activation in disordered graphites of the A_1_’ symmetry forbidden k ≠ 0 phonon: The origin of the D line. J. Chem. Phys..

[61] Ferrari A.C., Robertson J. (2000). Interpretation of Raman spectra of disordered and amorphous carbon. Phys. Rev. B.

[62] Tang L., Ji R., Cao X., Lin J., Jiang H., Li X., Teng K.S., Luk C.M., Zeng S., Hao J. (2012). Deep Ultraviolet Photoluminescence of Water-Soluble Self-Passivated Graphene Quantum Dots. ACS Nano.

[63] Peng J., Gao W., Gupta B.K., Liu Z., Romero-Aburto R., Ge L., Song L., Alemany L.B., Zhan X., Gao G. (2012). Graphene Quantum Dots Derived from Carbon Fibers. Nano Lett..

[64] Li Y., Hu Y., Zhao Y., Shi G., Deng L., Hou Y., Qu L. (2011). An Electrochemical Avenue to Green-Luminescent Graphene Quantum Dots as Potential Electron-Acceptors for Photovoltaics. Adv. Mater..

[65] Thaysen J.H., Thorn N.A. (1954). Excretion of Urea, Sodium, Potassium and Chloride in Human Tears. Am. J. Physiol.-Leg. Content.

[66] Baker L.B., De Chavez P.J.D., Ungaro C.T., Sopeña B.C., Nuccio R.P., Reimel A.J., Barnes K.A. (2019). Exercise intensity effects on total sweat electrolyte losses and regional vs. whole-body sweat [Na^+^], [Cl^−^], and [K^+^]. Eur. J. Appl. Physiol..

[67] Qu K., Wang J., Ren J., Qu X. (2013). Carbon Dots Prepared by Hydrothermal Treatment of Dopamine as an Effective Fluorescent Sensing Platform for the Label-Free Detection of Iron(III) Ions and Dopamine. Chem. A Eur. J..

[68] Ananthanarayanan A., Wang X., Routh P., Sana B., Lim S., Kim D.-H., Lim K.-H., Li J., Chen P. (2014). Facile Synthesis of Graphene Quantum Dots from 3D Graphene and their Application for Fe^3+^ Sensing. Adv. Funct. Mater..

[69] Edison T.N.J.I., Atchudan R., Shim J.-J., Kalimuthu S., Ahn B.-C., Lee Y.R. (2016). Turn-off fluorescence sensor for the detection of ferric ion in water using green synthesized N-doped carbon dots and its bio-imaging. J. Photochem. Photobiol. B Biol..

[70] Lai T., Zheng E., Chen L., Wang X., Kong L., You C., Ruan Y., Weng X. (2013). Hybrid carbon source for producing nitrogen-doped polymer nanodots: One-pot hydrothermal synthesis, fluorescence enhancement and highly selective detection of Fe(iii). Nanoscale.

[71] Wang D., Wang L., Dong X., Shi Z., Jin J. (2012). Chemically tailoring graphene oxides into fluorescent nanosheets for Fe^3+^ ion detection. Carbon.

[72] Qu S., Chen H., Zheng X., Cao J., Liu X. (2013). Ratiometric fluorescent nanosensor based on water soluble carbon nanodots with multiple sensing capacities. Nanoscale.

[73] Zhou L., Geng J., Liu B. (2013). Graphene Quantum Dots from Polycyclic Aromatic Hydrocarbon for Bioimaging and Sensing of Fe^3+^ and Hydrogen Peroxide. Part. Part. Syst. Charact..

[74] Annie Ho J.A., Chang H.-C., Su W.-T. (2012). DOPA-Mediated Reduction Allows the Facile Synthesis of Fluorescent Gold Nanoclusters for Use as Sensing Probes for Ferric Ions. Anal. Chem..

[75] Yu J., Xu C., Tian Z., Lin Y., Shi Z. (2016). Facilely synthesized N-doped carbon quantum dots with high fluorescent yield for sensing Fe^3+^. New J. Chem..

[76] Wei L., Ma Y., Shi X., Wang Y., Su X., Yu C., Xiang S., Xiao L., Chen B. (2017). Living cell intracellular temperature imaging with biocompatible dye-conjugated carbon dots. J. Mater. Chem. B.

