# Supplementary material for: The Role of N and S Doping on Photoluminescent Characteristics of Carbon Dots from Palm Bunches for Fluorimetric Sensing of Fe3+ Ion
Source: Int J Mol Sci. 2022 Apr 30;23(9):5001. doi: 10.3390/ijms23095001 (PMC9100793; doi:10.3390/ijms23095001)
Supplement: Supplementary file 1 [file ijms-23-05001-s001.zip › ijms-1680849-supplementary.pdf]

Supporting Information

The role of N/S doping on photoluminescent characteristics of carbon dots from palm bunches for fluorimetric sensing of Fe3+ ion

Aphinan Saengsrichan[1], Chaiwat Saikate[1], Peeranut Silasana[1], Pongtanawat Khemthong[2], Wanwitoo Wanmolee[2], Jakkapop Phanthasri[2], Saran Youngjan[2], Pattaraporn Posoknistakul[1], Sakhon Ratchahat[1], Navadol Laosiripojana[3], Kevin C.-W. Wu,[4-6] Chularat Sakdaronnarong[1,*]

[1] Department of Chemical Engineering, Faculty of Engineering, Mahidol University, 25/25 Putthamonthon 4 Road, Salaya, Putthamonthon, Nakhon Pathom 73170 Thailand

Tel: +66-28892138 ext. 6101-2, Fax: +662-44199731

[2] National Nanotechnology Center (NANOTEC), National Science and Technology Development Agency (NSTDA), Pathum Thani 12120, Thailand

[3] The Joint Graduate School of Energy and Environment, King Mongkut's University of Technology Thonburi, 126 Pracha Uthit Road, Bang Mot, Thung Khru, Bangkok 10140 Thailand

[4] Department of Chemical Engineering, National Taiwan University, No.1, Sec.4 Roosevelt Road, Taipei, 10617 Taiwan.

[5] Center of Atomic Initiative for New Materials (AI-MAT), National Taiwan University, Taipei 10617, Taiwan.

[6] International Graduate Program of Molecular Science and Technology, National Taiwan University (NTU-MST), Taipei 10617, Taiwan.

*Corresponding email address: chularat.sak@mahidol.ac.th

Table S1. Relative concentration of each chemical bond from deconvoluted peak from XPS measurement

Elements	CDs		US-CDs		PTS-CDs	
	Bonds	Atomic percent (%)	Bonds	Atomic percent (%)	Bonds	Atomic percent (%)
C 1s	O-C=O	51.99	C-C/C=C	25.05	C-C/C=C	13.08
	C-O	33.64	C-N/C-S	46.56	C-N/C-S	13.03
	C-C/C=C	14.35	C=O	22.27	C=O	26.42
			C=N	6.1	C=N	47.46
O 1s	C=O	58.2	C=O	24.52	C=O	45.16
	C-O-C	24.87	C-O-C	28.1	C-O-C	41.47
	O=C-O	16.92	O=C-O	47.37	O=C-O	13.36
N 1s			N=C	44.57	N=C	62.13
			C-N-C	30.63	C-N-C	29.43
			N-H	24.79	N-H	8.43
S 2p			C-SOx (1)	67.84	C-S-H	43.20
			C-SOx (2)	32.15	C-S-C	35.30
					C-SOx	21.48

Table S2. The element composition by XPS, average diameter from HRTEM, and average hydrodynamic diameter from zeta sizer of CDs, 0.05 M NS/CDs, 0.10 M NS/CDs, 0.20 M NS/CDs, 0.30 M NS/CDs, 0.40 M NS/CDs, CDs/PtNPs, and CDs/AgNPs

Materials	Atomic concentration (%)				Average diameter from HRTEM (nm)	Average hydrodynamic diameter from zeta sizer (nm)
	C 1s	O 1s	N 1s	S 2p		
CDs pure	70.68	27.25	2.06	ND	6.99 ± 1.69	1097.7 ± 352.0
0.05 M NS/CDs	70.06	24.97	2.58	2.38	9.46 ± 3.08	604.9 ± 169.7
0.10 M NS/CDs	76.65	18.34	7.06	2.96	4.48 ± 1.10	121.5 ± 31.5
0.20 M NS/CDs	66.37	25.9	4.2	3.53	4.91 ± 1.10	1265.2 ± 346.3
0.30 M NS/CDs	66.07	20.51	5.78	7.65	5.77 ± 0.90	2004.1 ± 541.8
0.40 M NS/CDs	63.66	26.49	3.49	6.36	20.41 ± 2.74	5931.7 ± 772.0

ND = not detected

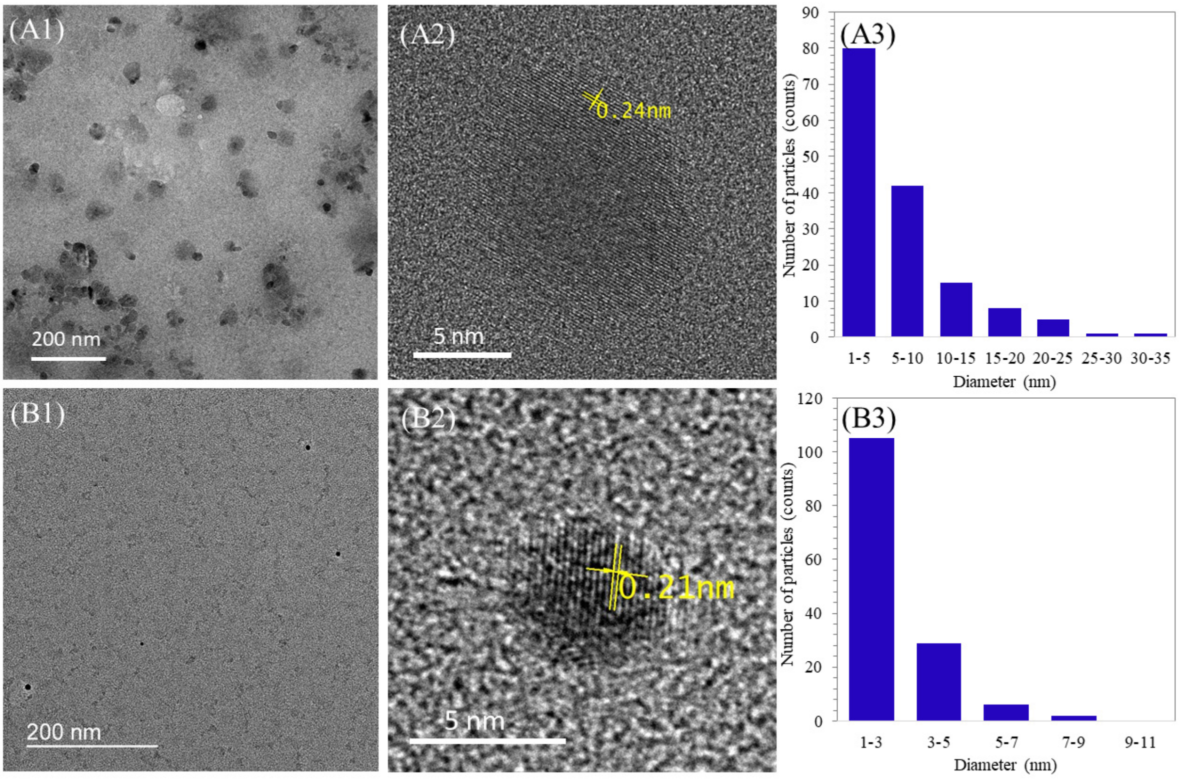

Figure S1 HRTEM images of synthesized CDs at ×120,000, ×300,000 magnifications and size distribution of (A) 220C 6 h EFB derived CDs, (B) 220C 10 h EFB derived CDs, (C) 220C 6H US-CDs, and (D) 220C 6H PNa-CDs.

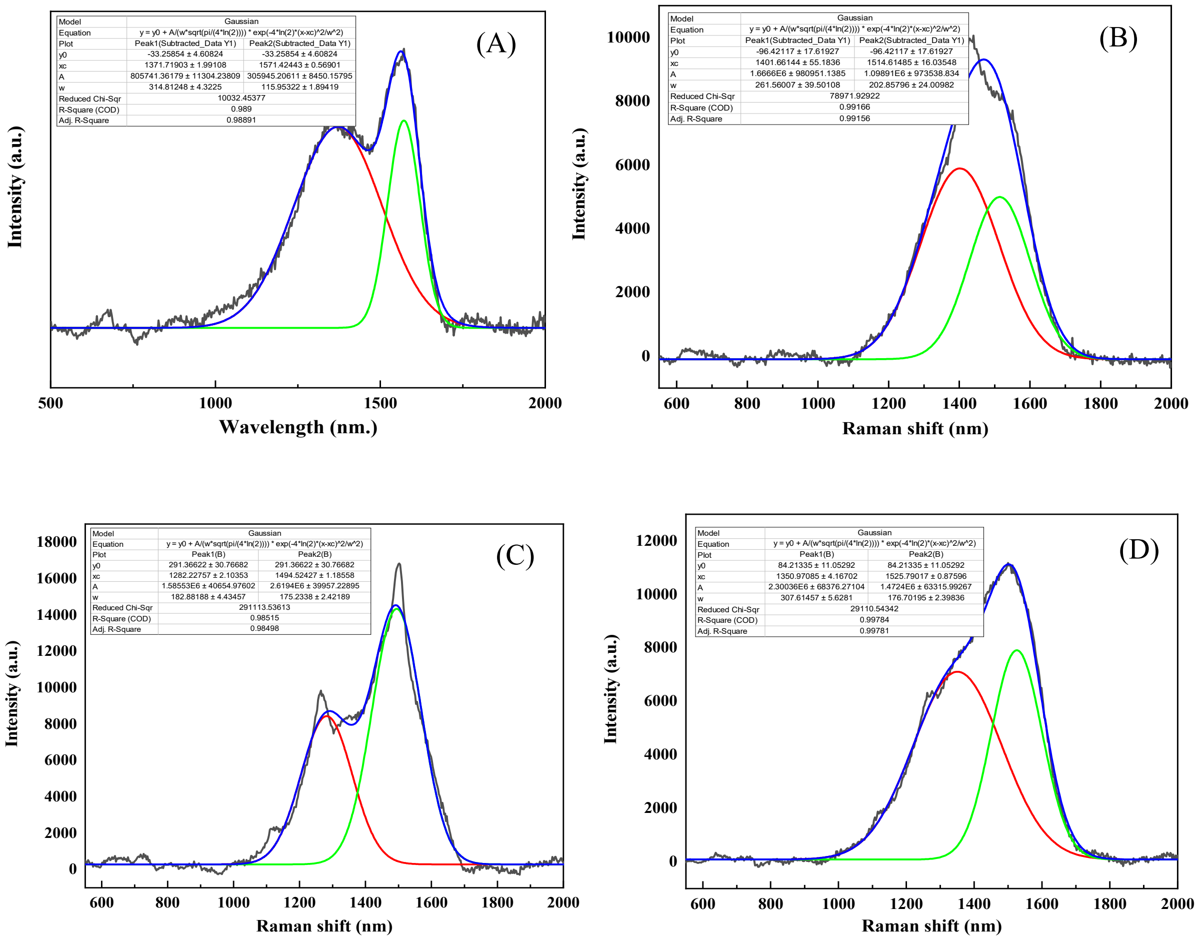

Figure S2 Deconvoluted Raman spectra for I_D/I_G ratio calculation of (A) CDs, (B) P-CDs, (C) PTS-CDs, and (D) PTS-CDs/Fe3+ (or 0.20 M NS/CDs)

Element	CDs	0.05 M NS/CDs	0.10 M NS/CDs	0.20 M NS/CDs	0.30 M NS/CDs	0.40 M NS/CDs
C 1s	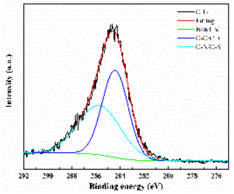	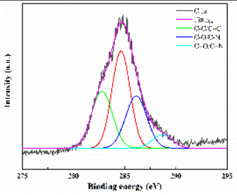	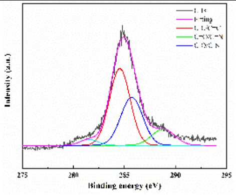	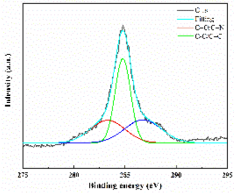	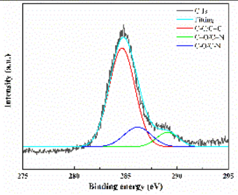	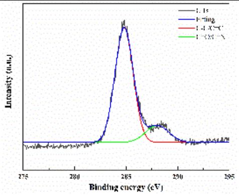
O 1s	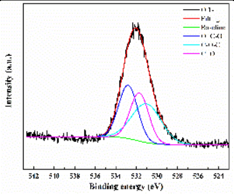	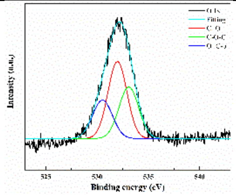	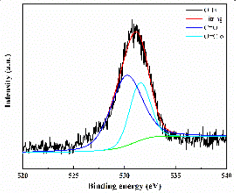	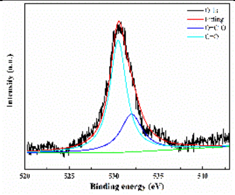	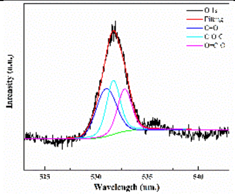	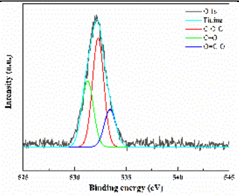
N 1s	-	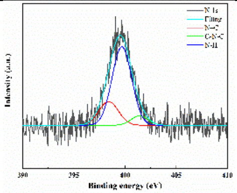	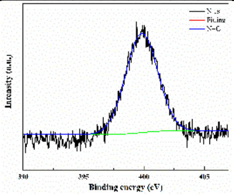	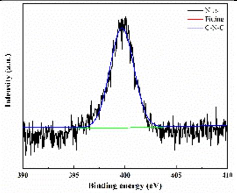	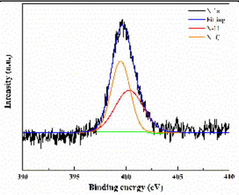	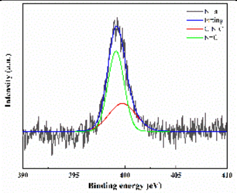
S 2p	-	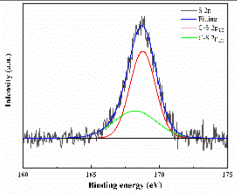	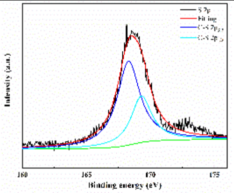	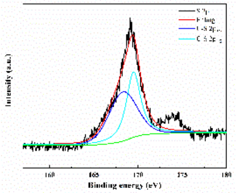	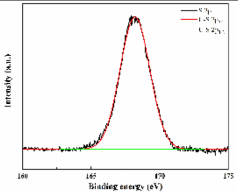	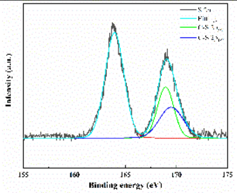

Figure S3. High resolution XPS spectra of CDs, 0.05M NS/CDs, 0.10M NS/CDs, 0.20M NS/CDs, 0.30M NS/CDs, and 0.40M NS/CDs for their C1s, O1s, N1s, and S2p
